# G protein-coupled receptor GPR182 negatively regulates sprouting angiogenesis via modulating CXCL12-CXCR4 axis signaling

**DOI:** 10.1007/s10456-025-09977-5

**Published:** 2025-05-02

**Authors:** Changsheng Chen, Wei Liu, Fang Yuan, Xiaoning Wang, Xi Xu, Chang Chun Ling, Xiaojuan Ge, Xiaozhong Shen, Bowen Li, Yuqian Shen, Dong Liu

**Affiliations:** 1 School of Life Sciences, Nantong Laboratory of Development and Diseases, Nantong University, Seyuan Road 9, Nantong, Jiangsu Province 226019 China; 2 Medical College of Nantong University, Nantong, Jiangsu Province China; 3 Research Center of Clinical Medicine, Affiliated Hospital of Nantong University, Nantong, Jiangsu Province China; 4 Department of Intervention and Vascular Surgery, Affiliated Hospital of Nantong University, Nantong, Jiangsu Province China; 5 State Key Laboratory of Medical Genomics, Shanghai Institute of Hematology, National Research Center for Translational Medicine, Ruijin Hospital Affiliated to Shanghai Jiao Tong University School of Medicine, Shanghai, China; 6 The State Key Laboratory Breeding Base of Basic Science of Stomatology & Key Laboratory of Oral Biomedicine Ministry of Education, School & Hospital of Stomatology, Medical Research Institute, Wuhan University, Wuhan, Hubei Province China; 7 Frontier Science Center for Immunology and Metabolism, Wuhan University, Wuhan, Hubei Province China; 8 Huai’an TCM Hospital Affiliated to Nanjing University of Chinese Medicine, Huai’an, Jiangsu Province China; 9 Department of Translational Medicine, IGBMC, INSERM U964, CNRS UMR7104, Université de Strasbourg, Illkirch, France; 10 Key Laboratory of Neuroregeneration of Jiangsu and Ministry of Education, Co-Innovation Center of Neuroregeneration, Nantong University, Nantong, Jiangsu Province China

## Abstract

Angiogenesis is a critical process for tumor progression, regulated by various signaling pathways. Although antiangiogenic therapies targeting the VEGF pathway have shown potential, their effectiveness is inconsistent across different tumor types. GPR182, an endothelial cell-specific G protein-coupled receptor, is frequently downregulated in hypervascular tumors, but its specific role in angiogenesis has not been well defined. Our study reveals that GPR182 expression is markedly reduced in hepatocellular carcinoma (HCC) and inversely correlates with CD31, a pan-endothelial marker. In zebrafish embryos, Gpr182 deficiency resulted in enhanced angiogenic sprouting and hypervascularization, and GPR182-deficient human umbilical vein endothelial cells (HUVECs) showed increased migration and proliferation. At the molecular level, GPR182 acts as a decoy receptor, binding CXCL12 and regulating its gradient, which in turn suppresses CXCR4-mediated angiogenesis. The pharmacological blockade of CXCR4 with AMD3100 corrected the abnormal angiogenic phenotype in Gpr182-deficient zebrafish embryos and in the livers of a zebrafish HCC model. This work uncovers GPR182 as a negative regulator of angiogenesis, a key process in tumor growth and metastasis, and proposes that targeting GPR182 may offer a novel therapeutic approach for antiangiogenic strategies in cancer treatment.

**Supplementary Information:**

The online version contains supplementary material available at 10.1007/s10456-025-09977-5.

## Introduction

Angiogenesis, the formation of new blood vessels from pre-existing ones, is crucial for both embryonic development and tumor progression. Hypervascular tumors, such as liver cancer, often demonstrate intense angiogenic activity, fueled by pro-angiogenic factors including VEGF, FGF, and angiopoietins [1]. The dense and chaotic vascular network in these tumors can impede the efficacy of therapies like chemotherapy and radiation, as they may not adequately penetrate the tumor. Targeting angiogenesis is a strategic approach in combating hypervascular tumors. Drugs that inhibit this process, such as bevacizumab—a monoclonal antibody against VEGF—can normalize tumor vasculature and curtail blood supply [2]. However, the clinical efficacy of VEGF-targeted therapies has been mixed, underscoring the need to identify new regulators of angiogenesis for treating hypervascular tumors.

G protein-coupled receptors (GPCRs) constitute a large family of membrane receptors that play pivotal roles in signal transduction across eukaryotic cells. Characterized by a seven-transmembrane domain structure, this diverse protein family encompasses over 800 members, categorized into five main classes [3]. GPCRs are associated with numerous human diseases, and more than 30% of marketed drugs target these receptors [4]. Despite the long-standing identification of many GPCRs, a significant portion remains orphan receptors with unknown functions [5].

GPR182, a member of the class A GPCR family, was initially identified as a candidate adrenomedullin receptor [6]. It was later classified as an orphan GPCR until recent findings revealed its role as a chemokine receptor, capable of binding chemokines such as CXCL10, CXCL12, and CXCL13 [7, 8]. *Gpr182* was found to be preferentially expressed in developing murine and zebrafish vascular endothelium during embryogenesis [9, 10]. Intriguingly, data from The Cancer Genome Atlas (TCGA) (https://www.cancer.gov/tcga) suggest that reduced *GPR182* expression is associated with hypervascular tumors. However, the precise mechanisms by which GPR182 contributes to tumor angiogenesis remain to be elucidated.

To elucidate the function of GPR182 in vascular development, we employed the zebrafish model organism, which presents distinct advantages over traditional mammalian models for angiogenesis research [11, 12]. The external development of zebrafish embryos post-fertilization facilitates direct, noninvasive observation of vascularization during embryogenesis. Their optical transparency is ideal for high-resolution imaging studies. Furthermore, zebrafish are amenable to forward genetic screens and genetic manipulation through morpholino oligonucleotide (MO) injections or CRISPR/Cas9 genome editing, providing a robust platform for studying gene function. A critical advantage of the zebrafish model is the dispensability of its circulatory system during the initial 5–7 days of development, which permits the study of genes that may be lethal when knockout or knockdown in mammalian systems. Additionally, the zebrafish vasculature development exhibits a high degree of similarity to that of mammals [13]. Employing the zebrafish model is thus instrumental in uncovering the mechanisms of angiogenesis.

In this study, we discovered a significant reduction of *GPR182* expression in hepatocellular carcinoma (HCC) samples and in the livers of a zebrafish HCC model. Loss-of-function in Gpr182 in zebrafish embryos led to excessive sprouting angiogenesis, attributed to enhanced EC migration and proliferation. RNA transcriptomic analysis revealed an upregulation of the chemokine receptor *cxcr4a* in the absence of Gpr182, implicating GPR182 in the modulation of CXCR4 signaling. However, GPR182, despite binding to its cognate chemokine CXCL12, does not initiate the classical signaling cascade or elicit the downstream cellular responses typical of conventional GPCRs, classifying it as an atypical chemokine receptor. Mechanistically, GPR182 competes with CXCR4 for CXCL12 binding, functioning as a decoy receptor that promotes the internalization and degradation of CXCL12. This action subsequently dampens the angiogenic CXCR4/CXCL12 signaling axis, preventing the sprouting of tip cells. Our findings unveil a novel mechanism in the regulation of angiogenesis and suggest GPR182 as a promising target for anti-angiogenic therapy and drug development.

## Results

### *GPR182* is underexpressed in HCC and inversely associated with poor prognosis

**Fig. 1:**
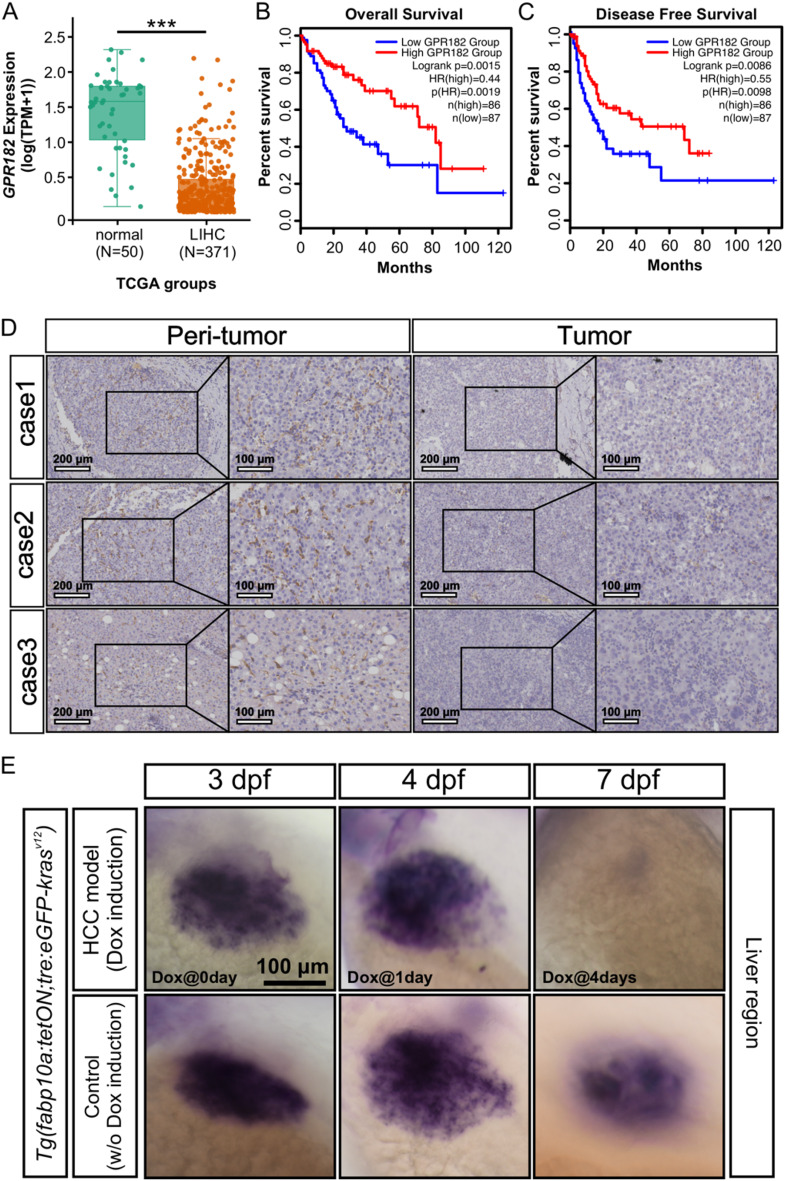
Downregulation of *GPR182* in HCC correlates with poor prognosis. **(A)** Comparative expression analysis of *GPR182* in normal liver versus HCC tissues within the TCGA-LIHC dataset. **(B** and **C)** Kaplan-Meier survival curves for overall survival (OS) and disease-free survival (DFS) of HCC patients with high and low expression levels of *GPR182* in TCGA-LIHC dataset. **(D)** Representative IHC staining for GPR182 in peritumoral and tumoral regions of HCC tissues. **(E)** Whole-mount in situ hybridization analysis of *gpr182* expression in zebrafish HCC models at various stages of tumor progression and in controls during development

We first analyzed *GPR182* expression levels in patients with HCC using publicly available data from TCGA. Our analysis revealed that *GPR182* expression was significantly lower in liver hepatocellular carcinoma (LIHC) compared to normal tissues (Fig. 1A). Additionally, high *GPR182* expression correlated with improved overall survival (OS) and disease-free survival (DFS) (Figs. 1B and C). To further validate these findings, we performed immunohistochemistry (IHC) on HCC samples, which demonstrated that GPR182 was barely detectable in the tumoral region compared to the peri-tumoral area (Fig. 1D). Next, we examined the spatio-temporal expression pattern of *gpr182* during HCC progression within zebrafish model using whole-mount in situ hybridization (WISH). The zebrafish HCC model was established using the transgenic line *Tg(fabp10a: tetON; tre: eGFP-kras*^*v12*^*)*, where the kras oncogene is specifically activated in hepatocytes upon doxycycline (DOX) induction [14]. Tumor induction began at 3 days post-fertilization (dpf), and *gpr182* expression was assessed at 0-, 1-, 2-, and 4-days post-induction (dpi) (Fig. 1E). Our results indicate a marked reduction in *gpr182* expression in the liver of the zebrafish HCC model, with levels progressively decreasing as tumor progression advanced (Fig. 1E).

### GPR182 is highly enriched in ECs but negatively correlated with EC marker CD31

**Fig. 2:**
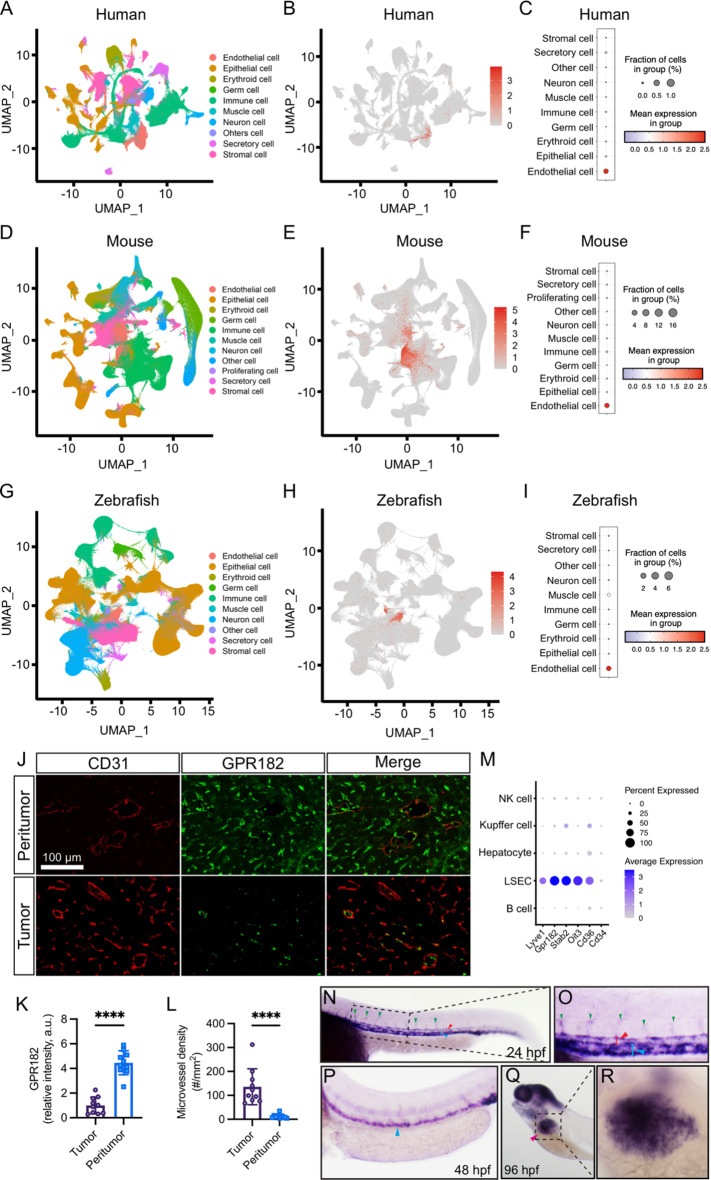
Comparative expression of GPR182 across species. **(A**,** D**,** G)** UMAP visualizations depict the distribution of GPR182 in the Human, Mouse, and Zebrafish cell atlases, respectively. **(B**,** E**,** H)** Relative distribution patterns of GPR182 across various cell clusters in Human, Mouse, and Zebrafish. **(C**,** F**,** I)** Mean expression levels of GPR182 within distinct cell clusters for each species. **(J)** Immunofluorescence staining of peritumoral and tumoral regions in HCC sections showing expressions of CD31 (a pan-endothelial marker) and GPR182. **(K** and **L)** Quantitative analysis of GPR182 expression intensity and microvessel density in peritumoral and tumoral regions of HCC sections (*n* = 10). Data are presented as mean ± SD, with statistical significance determined by Student’s t-test. ****, *p* < 0.0001. **(M)** Expression profiling of LSEC-specific markers in liver cell populations. **(N-R)** Whole-mount in situ hybridization of embryos reveals high *gpr182* expression in the zebrafish vascular system and liver. The hybridization signals in developing ISVs, PCV, DA, and liver are indicated by green, blue, red, and magenta arrowheads, respectively

To characterize the expression pattern of *GPR182* in different species, we reanalyzed single-cell RNA sequencing (scRNA-seq) datasets from human (GSE134355), mouse (GSE198832), and zebrafish samples (GSE198832) [15, 16]. Unsupervised dimensionality reduction and graph-based clustering analysis were performed with the datasets, and distinct cell clusters were visualized by the UMAP method (Fig. 2A, D, and G). *GPR182* gene was found to be highly enriched and expressed in EC clusters in all species (Fig. 2A-I). We further explored the relationship between GPR182 and the continuous EC marker CD31 in HCC tissues through immunofluorescence and immunohistochemistry (Fig. 2J and K). The results showed that CD31 expression was elevated in tumoral regions where GPR182 was seldom observed (Fig. 2J). Conversely, GPR182 was highly expressed in peritumoral areas, inversely correlating with blood vessel density (Fig. 2K and L). Additionally, through the analysis of single-cell RNA sequencing data (GSE47067) derived from mouse tissues [17], we observed that *Gpr182* is highly expressed in liver sinusoidal endothelial cells (LSECs), exhibiting a similar expression pattern to other LSEC-specific markers, including *Oit3*, *Lyve1*, *Cd36*, and *Stab2* (Fig. 2M). Whole-mount in situ hybridization (WISH) assays confirmed that zebrafish *gpr182* was expressed throughout the vascular system during embryogenesis (Fig. 2N-R). Notably, *gpr182* expression was observed in the dorsal aorta (DA) and posterior cardinal vein (PCV) at 24 h post fertilization (hpf), with sustained high levels in the PCV up to 48 hpf (Fig. 2N-P). The hybridization signal was apparently displayed in developing intersegmental vessels (ISVs) from 24 hpf onwards, with a pronounced concentration in the ventral half, diminishing by 48 hpf (Fig. 2N-P). At the later stage of zebrafish embryogenesis, *gpr182* mRNA was evident in the liver (Fig. 2Q and R). The temporal and spatial expression of *gpr182* in the zebrafish vascular system encompasses many key steps in embryonic angiogenesis [13], suggesting its regulatory role in angiogenesis.

### Loss-of-function in Gpr182 promotes sprouting angiogenesis

**Fig. 3:**
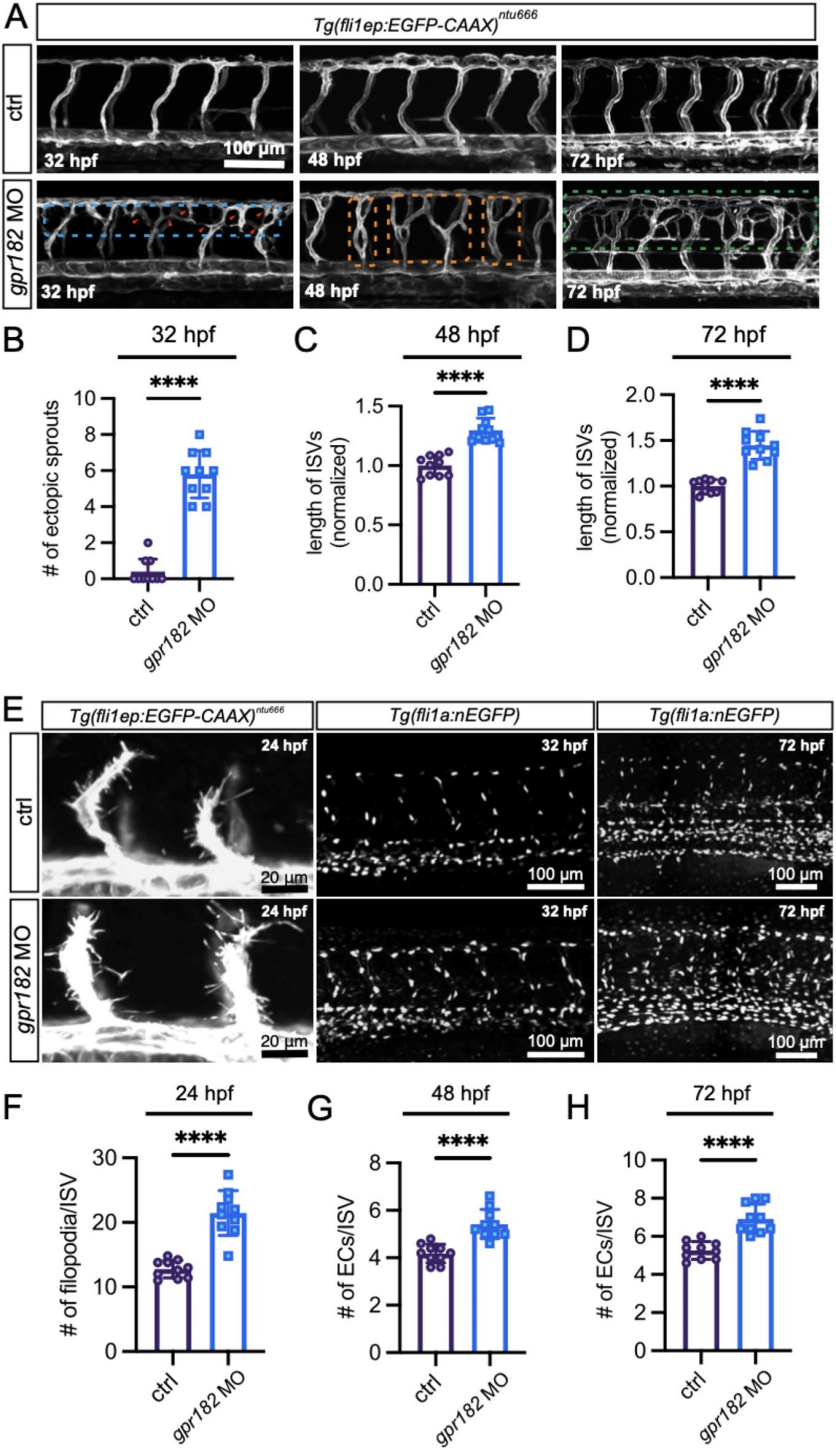
Gpr182 deficiency promotes sprouting angiogenesis in zebrafish embryos. **(A)** Trunk vascular morphology in control MO and *gpr182*-MO-injected *Tg(fli1ep: EGFP-CAAX)*^*ntu666*^ embryos at 32, 48, and 72 hpf. Gpr182 deficiency results in increased ISV sprouting. Dashed rectangles highlight aberrant vascular phenotypes, and red arrowheads point to the sprouts. Scale bar, 100 μm. **(B-D)** Quantitative analysis of ectopic sprouts at 32 hpf and total ISV length at 48 and 72 hpf. **(E)** Confocal microscopy analysis of ISV tip cell filopodia in 24-hpf *Tg(fli1ep: EGFP-CAAX)*^*ntu666*^ embryos and ISV endothelial cells in 32- and 72-hpf *Tg(fli1a: nEGFP)* embryos. **(F)** Number of ISV tip cell filopodia per ISV in control and *gpr182* morphant embryos at 24 hpf. **(G** and **H)** Number of ECs per ISV in control and *gpr182* morphant embryos at 32 and 72 hpf, respectively. Data are presented as mean ± SD, with each data point representing an individual fish. A total of 10 fish were analyzed per group. Statistical significance was determined using Student’s t-test. ****, *p* < 0.0001

To characterize the role of Gpr182 in sprouting angiogenesis, a splicing blocking MO-mediated knockdown was utilized to downregulate *gpr182* expression in the transgenic line *Tg(fli1ep: EGFP-CAAX)*^*ntu666*^, in which a chimera endothelial enhancer/promoter fragment (fli1ep) was employed to drive the specific expression of EGFP in ECs, and the CAAX membrane targeting motif enabled the localization of EGFP at the EC surface. Zebrafish harbor a single ortholog of the human *GPR182* gene, with high sequence homology and conserved protein domains, as demonstrated through multiple alignments and structural predictions (Supplementary Fig. 1). This conservation suggests a preserved functional role across species. In vivo confocal imaging of the trunk vasculature in the *gpr182* morphants of *Tg(fli1ep: EGFP-CAAX)*^*ntu666*^ zebrafish revealed abnormal angiogenic sprouts emerging from the dorsal aspect of intersegmental vessels (ISVs) or dorsal longitudinal anastomotic vessels (DLAVs) at 32 hpf (Fig. 3A). The excessive growing blood vessels anastomosed with ISVs or DLAVs at 48 hpf and evolved into a complex vascular network at 72 hpf without vascular retraction (Fig. 3A). Various aberrant vascular phenotypes were observed in *gpr182* morphants, including Y-shaped structure formed by a single ISV gave rise to two sprouts connected to DLAV, H-shaped structure formed by an additional sprout connected two neighbor ISVs laterally, and a parallel ISV formed in close proximity to the pre-existing one (Fig. 3A). Compared to control siblings, these morphants displayed an increased number of ectopic sprouts and elongated ISVs in the trunk region (Fig. 3B-D). Importantly, the hyperbranching vascular phenotype was rescued by overexpressing *gpr182* (Supplementary Fig. 2).

During the process of vascular pruning and remodeling, excessive blood vessels typically undergo retraction if they lack blood flow. However, in our study, we observed that these abnormally formed vessels persisted post-remodeling at 72 hpf. To investigate this phenomenon, we assessed blood flow in embryos harboring erythrocyte-labeled gata1:DsRed reporter and discovered that these aberrant vessels were not only lumenized but also perfused with blood (Supplementary Fig. 3A). Moreover, the aberrant vascular phenotypes could be recapitulated within *gpr182* morpholino-injected *Tg(flt1*^*BAC*^:*YFP); Tg(kdrl: ras-mCherry)* double transgenic embryos, in which the arterial vessels are labeled with YFP and the whole vasculature are labeled with mCherry (Supplementary Fig. 3B), excluding a potential mistargeting effect of *gpr182* morpholino.

To evaluate the effects of Gpr182 loss-of-function on EC behaviors, we conducted a detailed analysis of endothelial tip cell migration in 24-hpf embryos of *Tg(fli1ep: EGFP-CAAX)*^*ntu666*^, as well as stalk cell proliferation in 32 and 72 hpf embryos of *Tg(fli1a: nEGFP)* (Fig. 3E). Our findings revealed a significant increase in the number of filopodia in primary angiogenic sprouts at 24 hpf and in endothelial cell counts per ISV at both 32 and 72 hpf in the *gpr182* morphants compared to control groups (Fig. 3F-H).

**Fig. 4:**
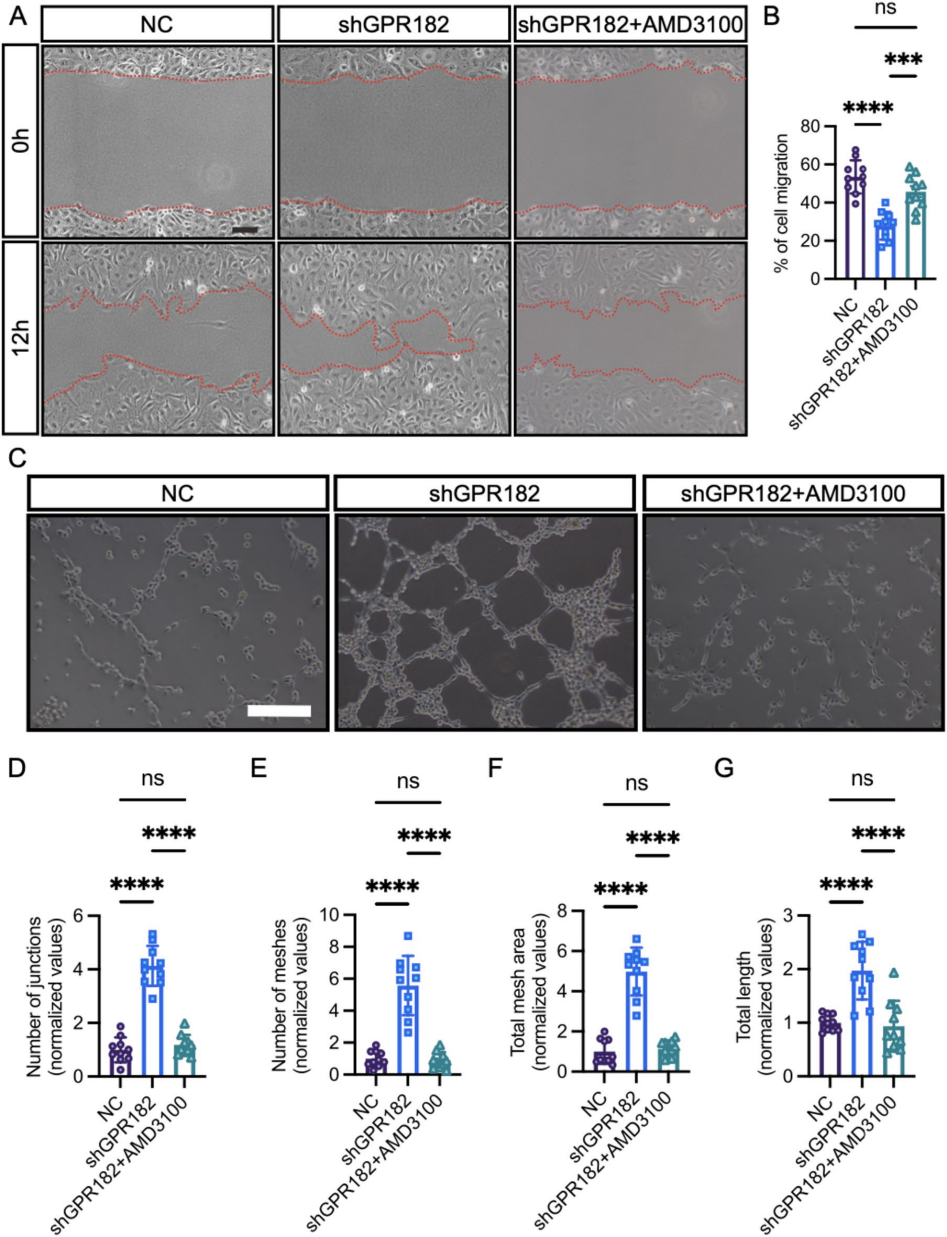
Loss of GPR182 promotes EC migration and tube formation. **(A** and **B)** EC migration is assessed using the wound closure assay in HUVECs. Scale bar, 100 μm. **(C)** Representative microscopic images display tube formation by HUVECs. Scale bar, 100 μm. **(D-G)** Quantitative analysis of the number of junctions, meshes, total mesh area, and total tube length in HUVECs. Data are presented as mean ± SD. Statistical significance was determined using Student’s t-test. ****, *p* < 0.0001

To substantiate the impact of GPR182 on angiogenesis, we utilized lentiviral transduction to knock down GPR182 in HUVECs. Our in vitro assays revealed that the knockdown of GPR182 significantly enhanced HUVEC migration, as depicted in Fig. 4A and B. Concurrently, the ability of these cells to form tubes was markedly elevated following *GPR182* knockdown, characterized by an increase in the number of junctions and meshes, as well as the total mesh area and tube length of the vascular network (Fig. 4C-G).

These results collectively highlight a novel regulatory role for GPR182 in sprouting angiogenesis. The loss of GPR182 function stimulates EC migration and proliferation in both in vivo and in vitro settings, thereby driving the process of sprouting angiogenesis.

### Gpr182 loss-of-function leads to enhancement of *cxcr4a* expression

**Fig. 5:**
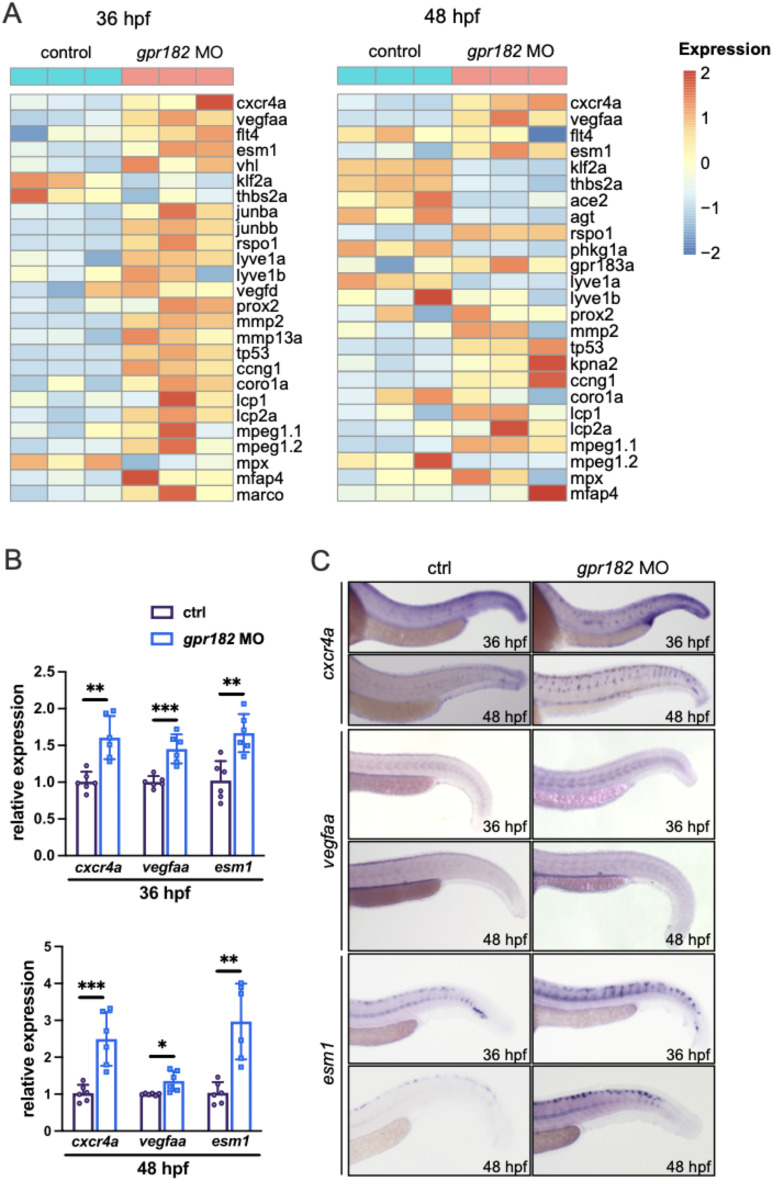
Whole-genome transcriptomic profiling of control embryos and *gpr182* morphants. **(A)** Heatmaps based on bulk RNA-seq data represent the expressions of cardiovascular-related genes in 36 and 48 hpf embryos. Zebrafish *cxcr4a*, *vegfaa*, and *esm1* are upregulated in *gpr182* morphants. **(B** and **C)** Expression levels of *cxcr4a*, *vegfaa*, and *esm1* are validated by qPCR and WISH. Data are presented as mean ± SD, with statistical significance determined by Student’s t-test. *, *p* < 0.05; **, *p* < 0.01; ***, *p* < 0.001

To uncover the molecular mechanisms by which Gpr182 deficiency promotes sprouting angiogenesis, we conducted a comprehensive analysis of gene expression changes in *gpr182* knockdown embryos at 36 and 48 hpf using bulk RNA transcriptomic profiling (Fig. 5A). Our analysis revealed significant alterations in a suite of genes pivotal to angiogenesis. Notably, the chemokine receptor *cxcr4a*, recognized for its role as an endothelial tip cell marker and a key promoter of angiogenic sprouting [18, 19], was markedly upregulated in the absence of Gpr182 (Fig. 5A). Furthermore, the expression of *vegfaa*, a principal regulator of angiogenesis, and *esm1*, another tip cell marker, was also elevated in the Gpr182 loss-of-function scenario (Fig. 5A). The expression of *cxcr4a*, *vegfaa*, and *esm1* was verified by qPCR and WISH assay, respectively (Fig. 5A-B). Additionally, treatment of shGPR182-transfected HUVECs with AMD3100, a CXCR4 signaling inhibitor [20, 21], effectively restored the abnormal phenotype resulting from GPR182 loss of function (Fig. 4). These findings prompt the hypothesis that GPR182 may modulate sprouting angiogenesis through a CXCR4-dependent mechanism.

### GPR182 lacks intracellular G protein-mediated downstream signaling in response to ligand binding

CXCR4, a prototypical chemokine receptor, is typically modulated by atypical chemokine receptors (ACKRs), such as ACKR3 (also known as CXCR7) [22, 23]. These ACKRs, while structurally similar to GPCRs, lack the DRYLAIV motif necessary for G protein coupling and subsequent intracellular signaling [24]. Human GPR182 (hGPR182), akin to ACKR3, is missing this motif, implying potential atypical signaling characteristics. However, whether GPR182 acts as an ACKR that fails to initiate G protein-mediated signaling remains a subject of debate [8, 25]. To elucidate the signaling mechanisms of GPR182, we employed a live-cell fluorescence imaging system designed to monitor the dynamics of GPCR downstream signaling [26]. This system utilized two stable Hela cell lines genetically engineered to express biosensors for real-time detection of cAMP, Ca^2+^, RhoA, and ERK signals. These cell lines were co-transfected with hGPR182 expression plasmids and selected using puromycin to isolate GPCR-expressing cells.

**Fig. 6:**
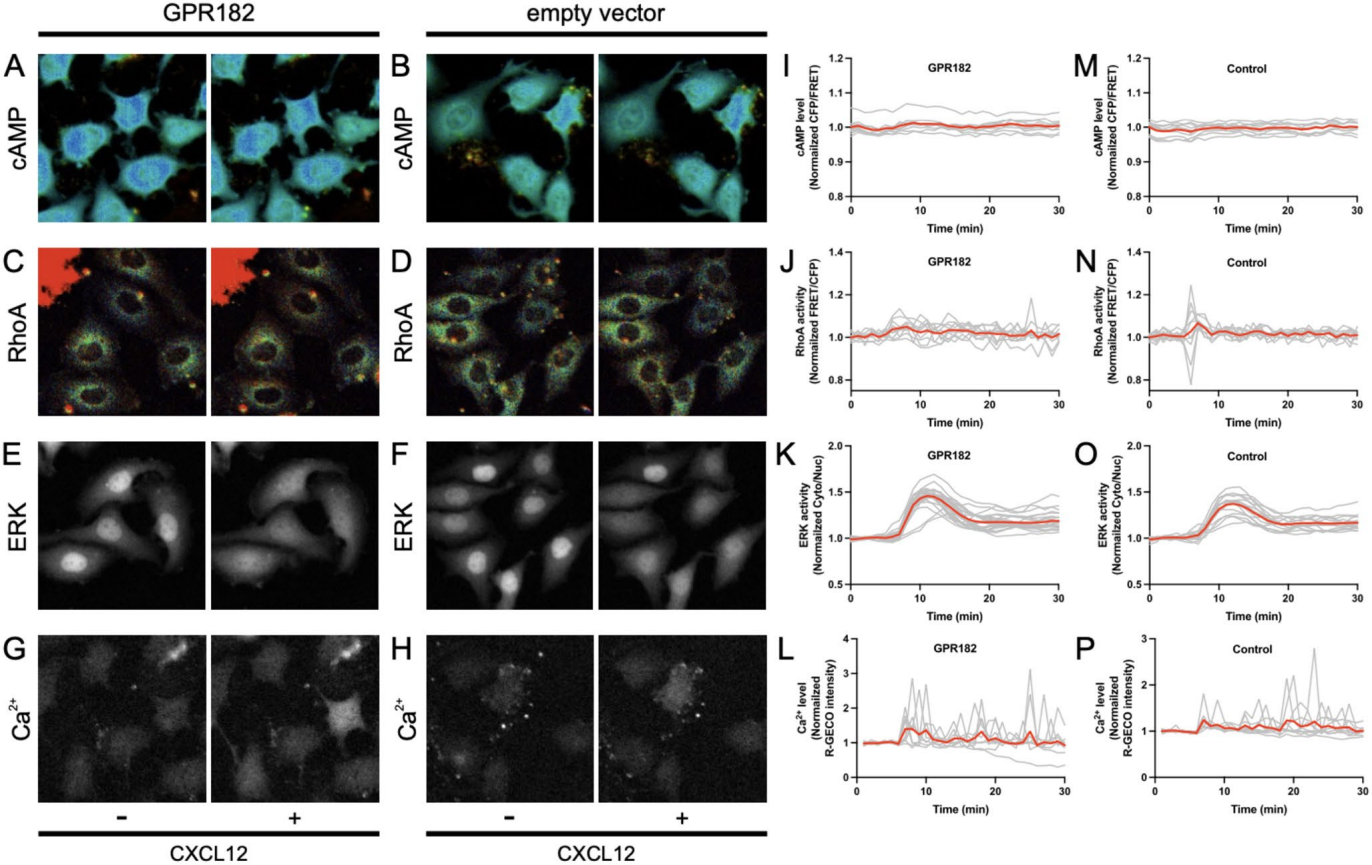
GPR182 does not trigger downstream signaling in response to CXCL12. **(A** and **G)** and **(B** and **H)**, HeLa/cAMP/Ca2 + cells expressing GPR182 or an empty vector were treated with CXCL12. **(C** and **D)** and **(E** and **F)**, HeLa/RhoA/ERK cells expressing GPR182 or an empty vector were treated with CXCL12. The addition CXCL12 starts at 5 min. Representative images of different biosensors before (*left*) and after (*right*) the stimulation of CXCL12. The response of the biosensors for cAMP levels **(I** and **M)**, RhoA activity **(J** and **N)**, ERK activity **(K** and **O)**, and Ca2 + levels **(L** and **P)** is normalized by dividing by the averaged value before stimulation and plotted as a function of elapsed time after stimulation. The red and gray lines represent the time-course for the average and individual cells, respectively

Using CXCL12, the specific ligand for CXCR4, we stimulated GPR182, as previous studies have shown high-affinity binding between CXCL12 and GPR182 [8]. The activation dynamics of cAMP, Ca^2+^, RhoA, and ERK in response to CXCL12 were quantified using the live-cell imaging system. HeLa cells with GPR182 expression showed no alteration in cAMP and RhoA signaling in response to CXCL12 stimulation, similar to cells lacking GPR182 expression (Fig. 6A-D). Despite elevated basal levels of ERK and Ca^2+^ in control cells, GPR182 expression did not augment these signals (Fig. 6E-H).

Our findings suggest that GPR182 fails to trigger downstream signaling cascades following CXCL12 stimulation, reinforcing its classification as an atypical chemokine receptor.

### GPR182 regulates CXCL12 bioavailability through chemokine scavenging

**Fig. 7:**
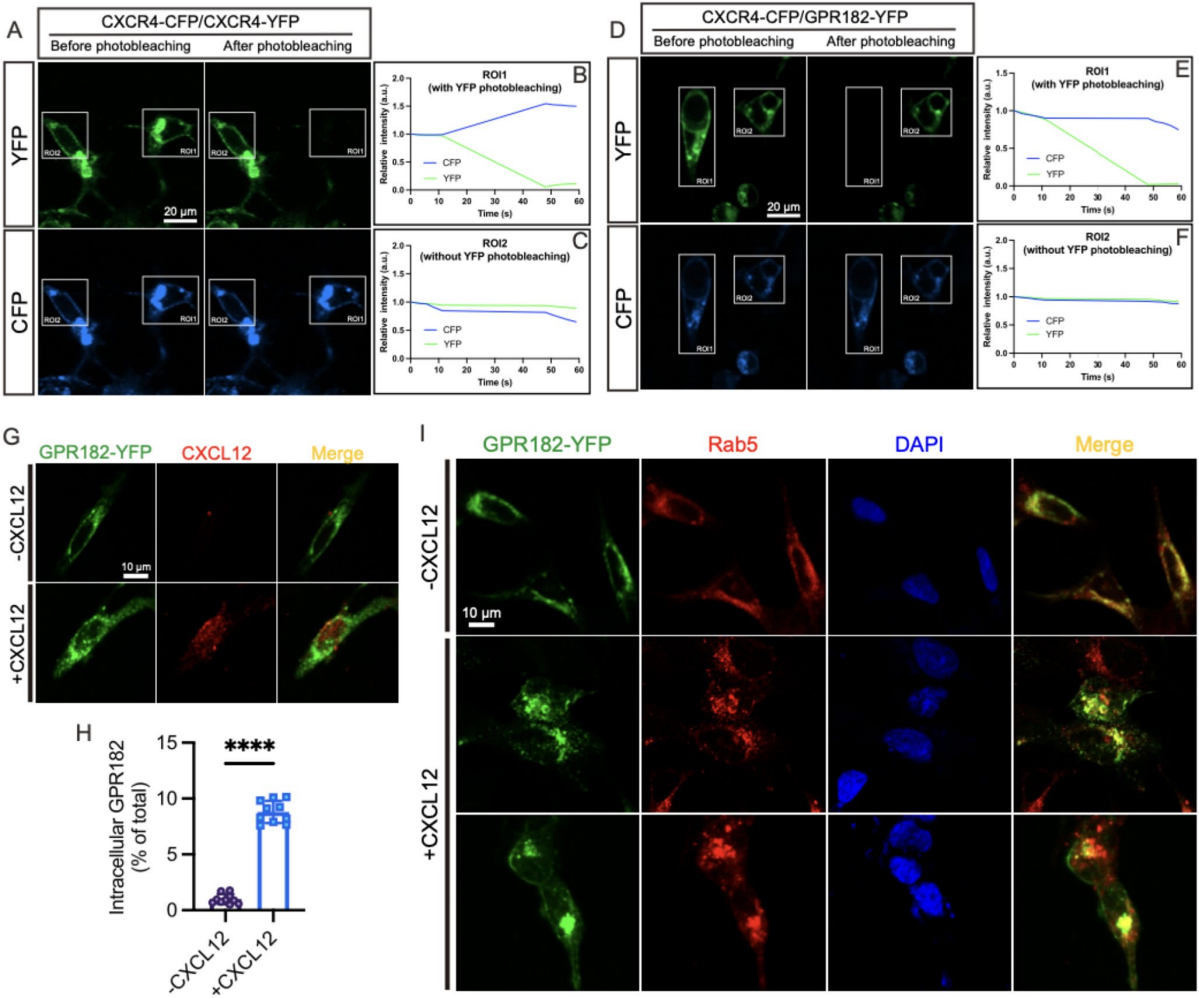
GPR182 modulates CXCR4 signaling through internalizing CXCL12. **(A** and **D)** FRET analyses via acceptor photobleaching assess the formation of CXCR4 homodimers (positive control) and CXCR4/GPR182 heterodimers. Representative images display CFP and YFP fluorescent signals before and after photobleaching. White rectangles indicate the regions of interest (ROI) for photobleaching; negative controls lack photobleaching. **(B**,** C**,** E**, and **F)** Relative intensities of CFP and YFP in the corresponding ROIs for panels A **(B** and **C)** and D **(E** and **F)**. **(G)** Confocal microscopy images depict CXCL12 internalization mediated by GPR182. HEK293T cells expressing YFP-tagged GPR182 are incubated with or without CXCL12, and endocytosis is assessed using an anti-CXCL12 antibody. **(H)** Quantification of intracellular GPR182 localization as shown in panel G. **(I)** Intracellular colocalization of GPR182 and Rab5 upon CXCL12 addition. HEK293T cells expressing YFP-tagged GPR182 are incubated with or without CXCL12, and cells are stained with an anti-Rab5 antibody

Our research uncovers that GPR182 functions as an ACKR that fails to trigger downstream G protein-mediated signaling upon chemokine binding. However, how does GPR182 regulate CXCL12/CXCR4 signaling remains unrecognized. As the closest paralogue of GPR182, ACKR3 adopts two strategies for regulating CXCL12-CXCR4 axis-mediated signaling: by heterodimerizing with CXCR4 to facilitate its internalization and degradation, and by acting as a decoy receptor for CXCL12 scavenging [23]. To explore the potential interaction between GPR182 and CXCR4, we utilized the Förster resonance energy transfer (FRET) assay. This technique enables the detection of receptor dimerization at the single-cell level. FRET occurs when two proteins tagged with a fluorescence donor and acceptor are in close proximity, typically within 100 Å, the maximum distance for protein-protein interaction. In our experiments, human CXCR4 (hCXCR4) and human GPR182 (hGPR182) were fused to the FRET donor CFP and acceptor YFP, respectively. The CFP-tagged hCXCR4 (hCXCR4-CFP) and YFP-tagged hGPR182 (hGPR182-YFP) were co-transfected into HEK293T cells and subjected to acceptor-photobleaching-based FRET analysis. If energy transfer occurs from the donor to the acceptor, the fluorescence intensity of the donor decreases. When the acceptor fluorophore is bleached, the energy transfer from donor to acceptor is prevented, and thus the fluorescence intensity of the donor increases. Figure 7A presents the confocal images of HEK293 cells coexpressing hCXCR4-CFP and hCXCR4-YFP. ROI1 underwent acceptor photobleaching, while ROI2 served as an untreated control. A robust FRET signal, indicative of hCXCR4-CFP and hCXCR4-YFP interaction, was observed in ROI1 (Fig. 7A and B, Table 1), thereby confirming the homodimerization of CXCR4 [27]. Furthermore, the addition of CXCL12 to the culture medium led to a modest enhancement in FRET signals between CXCR4 homodimers, as detailed in Supplementary Table 1. In the absence of acceptor photobleaching, the fluorescence intensity of CFP in ROI2 continuously diminished (Fig. 7A and C). In comparison, the FRET phenomenon between hCXCR4-CFP and hGPR182-YFP was absent (Fig. 7D-F). These results suggested that the deduction that GPR182 physically interacts with CXCR4 for receptor internalization and degradation is precluded.

To determine if GPR182 acts as a decoy receptor modulating CXCL12 levels, we tracked CXCL12 internalization in HEK293T cells expressing YFP-tagged hGPR182 (Fig. 7G). In the absence of CXCL12, GPR182 was primarily localized to the cell membrane (Fig. 7G). However, upon CXCL12 stimulation, the receptor translocated intracellularly accompanied by the chemokine (Fig. 7G-H). Further analysis of GPR182 co-localization with the endosome marker Rab5 following CXCL12 treatment revealed a concentrated cytoplasmic signal, indicating that GPR182’s intracellular translocation is mediated by endocytic trafficking (Fig. 7I).

**Table 1:**
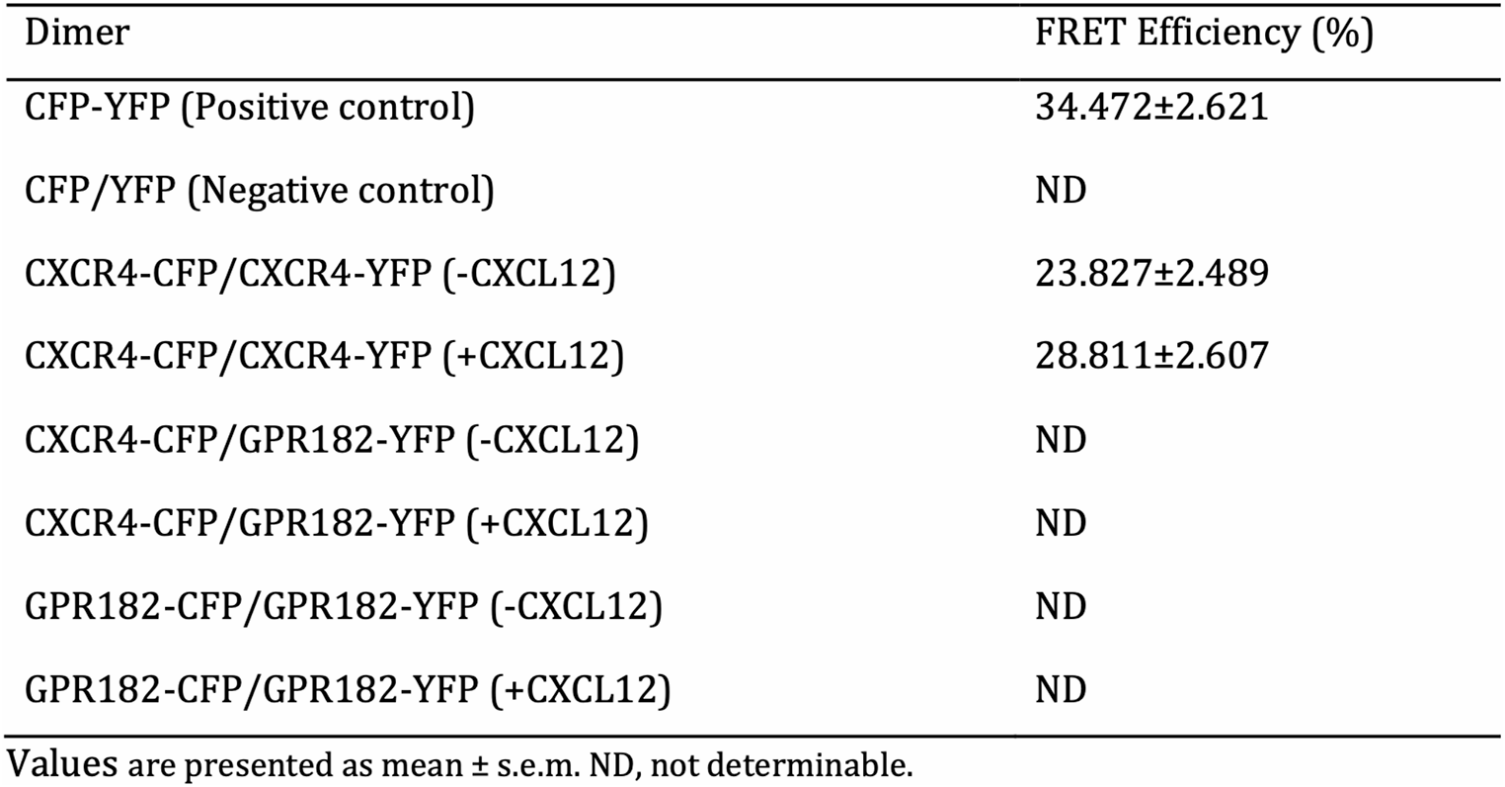
FRET analysis for CXCR4 and GPR182 homo- and heterodimers

### Inhibition of CXCR4 signaling normalizes the vasculature in HCC

**Fig. 8:**
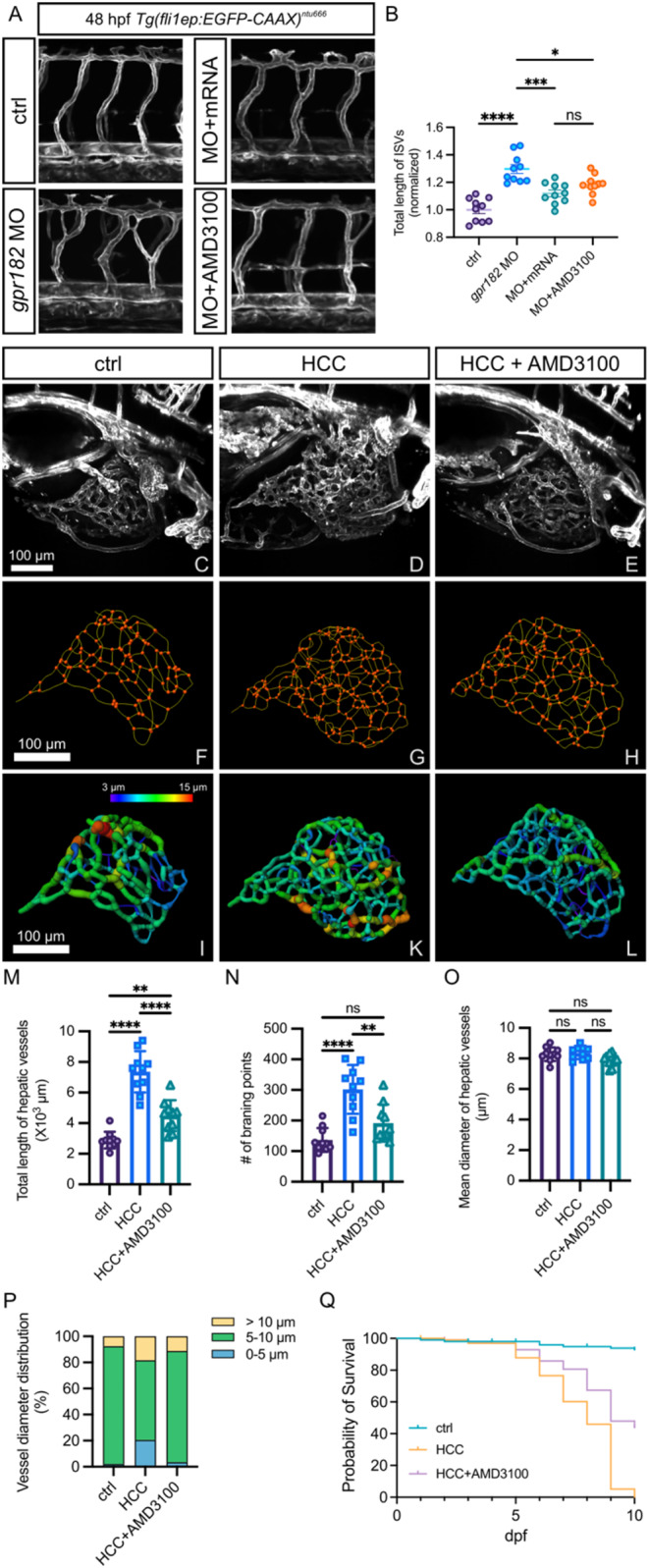
Inhibition of CXCR4 signaling normalizes the vasculature in *gpr182* morphants and the liver of HCC model. **(A)** Confocal microscopy images of trunk vessels in 48-hpf *Tg(fli1ep: EGFPCAAX)*^*ntu666*^ control embryos, embryos injected with *gpr182* MO, embryos injected with *gpr182* MO and *gpr182* mRNA, and embryos injected with *gpr182* MO and AMD3100. **(B)** Quantitative analysis of total ISV length. **(C-E)** Confocal images of liver vasculature in control zebrafish, HCC model zebrafish, and AMD3100-treated HCC zebrafish at 7 dpf. **(F-L)** Three-dimensional reconstructions of liver vasculature in controls, HCC zebrafish, and AMD3100-treated HCC zebrafish. Vascular vessels and branching points are indicated by green and orange, respectively. **(M-O)** Quantifications of total hepatic vessel length, number of vascular branching points, and vessel diameters of liver vasculature. Data are presented as mean ± SD. One-way ANOVA analysis was applied. Not significant (ns); ****, *p* < 0.0001. **(P)** Vessel diameter distribution in controls, HCC zebrafish, and AMD3100-treated HCC zebrafish. **(Q)** Survival rates of controls, HCC zebrafish, and AMD3100-treated HCC zebrafish until 10 dpf

To assess the functional relevance of *cxcr4a* upregulation in the excessive sprouting observed in *gpr182* morphants, we treated them with CXCL12/CXCR4 signaling inhibitor, AMD3100. Treatment with AMD3100 effectively mitigated the hyperbranching phenotype in the trunk vasculature of Gpr182-deficient embryos (Fig. 8A and B). Further, to explore the therapeutic potential of CXCR4 inhibition in HCC, we administered AMD3100 to the zebrafish HCC model and analyzed liver vascular changes. The HCC liver vasculature exhibited disorganized features such as complex networks, excessive branching, and irregular growth patterns compared to controls (Fig. 8C and D). To quantify the vascular parameters, we reconstructed the three-dimensional structure of the liver vasculature (Fig. 8F, G, I and K). Quantifications of the liver vasculature showed significant increases in total hepatic vessel length and vascular branching points (Fig. 8M and N), indicative of tumor neoangiogenesis during HCC progression. Although the mean diameter of the liver vessel in HCC was not different from that in controls (Fig. 8O), the HCC liver vasculature had more uneven lumen calibers (Fig. 8P). These morphological characteristics closely resemble the phenotype observed in human liver cancer [28]. Treatment with AMD3100 normalized the aberrant liver vasculature in the HCC model (Fig. 8E, H, and L-P). In addition, inhibiting CXCR4 signaling significantly improved the survival rate of HCC zebrafish (Fig. 8Q). These results suggest that targeting the CXCR4 signaling pathway could be a promising therapeutic strategy for vascular normalization in HCC.

## Discussion

Angiogenesis is fundamental to a variety of physiological and pathological activities. Physiological angiogenesis occurs rapidly but transiently and is tightly regulated, whereas pathological angiogenesis, which occurs during chronic inflammation and tumor growth, results from an imbalance between positive and negative angiogenic regulators [29]. Tumor angiogenesis, the formation of new blood vessels within a tumor, is critical for tumor growth, progression, and metastasis. Hypervascularization is a common phenotype in various cancers, including HCC and glioblastoma. Elucidating the unique features of hypervascular tumors and their regulatory mechanisms is vital for devising effective therapeutic strategies. Anti-angiogenic therapies, designed to deprive tumors of nutrients and oxygen, have made significant strides in cancer treatment, providing an alternative or complementary approach to conventional cytotoxic chemotherapy. However, treatments like bevacizumab, a humanized monoclonal antibody targeting VEGF, and small molecule inhibitors like sorafenib and sunitinib, which target VEGFR, have shown limited efficacy in cancers such as HCC. Beyond the classical VEGF signaling, G-protein coupled receptors (GPCRs) are also implicated in the regulation of physiological and pathological angiogenesis [30, 31]. Identifying novel regulators of angiogenesis is a critical step forward in the field of vascular biology and has significant implications for both basic science and clinical medicine. Novel regulators hold significant promise as therapeutic targets for diseases where angiogenesis plays a key role, such as various types of cancer and age-related macular degeneration. Furthermore, novel regulators may also function as biomarkers for the diagnosis and prognosis of angiogenesis-related diseases.

G protein-coupled receptor GPR182, a member of the Rhodopsin receptor family, was initially identified as a potential adrenomedullin receptor, although subsequent research has excluded this notion [6, 7]. GPR182 is predominantly expressed in the liver, heart, immune system, and ECs during embryonic development [25, 32]. Our study reveals that GPR182 expression is downregulated in HCC tissues and inversely correlates with microvessel density, aligning with data from the TCGA-LIHC dataset. These findings suggest a role for GPR182 in angiogenesis within tumor pathology. Utilizing a zebrafish model, we discovered that the zebrafish homologue *gpr182* is highly enriched in ECs of the vascular system. Knockdown of *gpr182* in zebrafish led to excessive sprouting angiogenesis and abnormal neovascularization during embryonic development. Both in vivo and in vitro studies indicated that GPR182 negatively regulates sprouting angiogenesis by modulating EC migration, proliferation, and tube formation. Differential gene expression analysis identified *cxcr4a* as significantly upregulated in *gpr182* morphants, among other known angiogenesis-related genes such as *vegfaa* and *esm1*. Notably, pharmacological inhibition of *cxcr4a* with AMD3100 normalized the hypervascularized phenotype, suggesting that GPR182 may regulate angiogenesis through the CXCR4 signaling pathway.

**Fig. 9:**
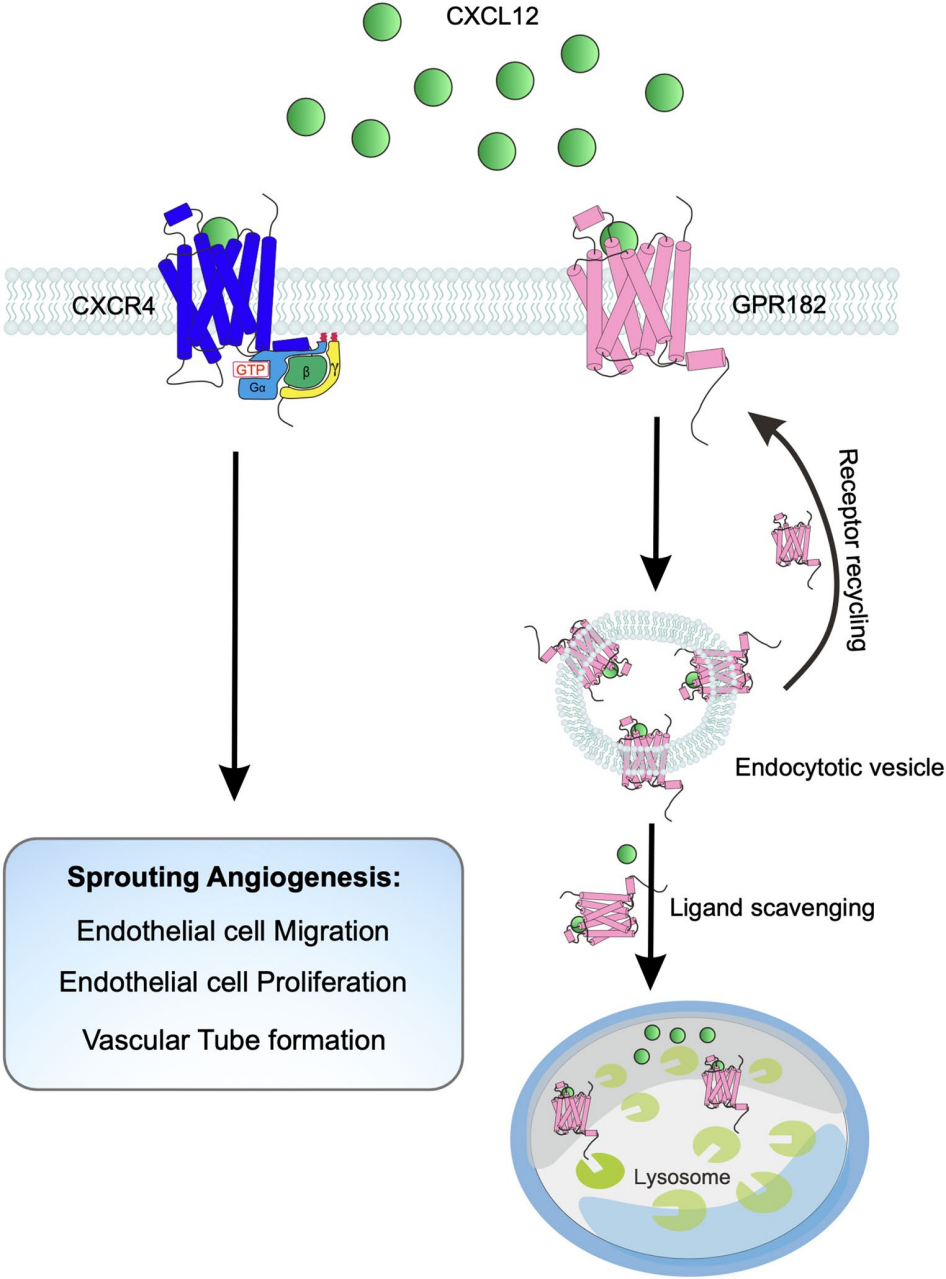
Schematic overview of GPR182’s role in regulating sprouting angiogenesis. This illustration depicts how GPR182 functions as a chemokine scavenger to modulate the CXCL12/CXCR4 signaling axis, thereby controlling the process of sprouting angiogenesis

The chemokine receptor CXCR4 and its ligand CXCL12 play pivotal roles in modulating cellular behaviors, including migration and proliferation, across a spectrum of developmental and pathological processes. These include immune cell homing, germ and neuronal cell migration, cardiac development, angiogenesis, lymphangiogenesis, and tumor progression [33–40]. Identified as an endothelial tip cell-enriched gene, CXCR4 is a key promoter of angiogenesis. Its inhibition leads to significant changes in tip cell morphology and vascular patterning, characterized by the disruption of interconnections between tip cells and a decrease in the vascularized area [18, 37]. Our study reveals that the loss of Gpr182 in zebrafish leads to increased expression of *cxcr4a*, a key chemokine receptor in angiogenesis. Despite this, the precise role of GPR182 in the CXCL12-CXCR4 signaling pathway remains elusive. Utilizing a state-of-the-art live-cell fluorescence imaging system to monitor GPCR downstream signaling dynamics, we discovered that GPR182 does not initiate downstream G protein-mediated signaling upon CXCL12 binding, affirming its classification as an atypical GPCR [26]. We propose two hypotheses for how GPR182 might regulate angiogenesis through the CXCR4 signaling axis: [1] GPR182 forms heterodimers with CXCR4, promoting the internalization and degradation of CXCR4, either dependently or independently of ligand binding; [2] GPR182 and CXCR4 compete for CXCL12 binding, with GPR182 serving as a decoy receptor that inhibits CXCR4/CXCL12-mediated signaling. FRET analysis showed no interaction between GPR182 and CXCR4, but a clear FRET signal was detected within the CXCR4/CXCR4 homodimer pair, indicating the uncoupling between GPR182 and CXCR4 and confirming the constitutive homodimerization of CXCR4 [27]. This finding rules out the hypothesis that GPR182 interacts with CXCR4 for receptor degradation. To explore the alternative hypothesis, we monitored the endocytosis of CXCL12. Our observations revealed that CXCL12 is sequestered in the cytoplasm, where it colocalizes with GPR182 and endosomes. This suggests that GPR182 functions as a scavenger receptor, regulating the gradient and bioavailability of CXCL12 (Fig. 9).

Our study uncovers a novel role for the EC-enriched G protein-coupled receptor GPR182 in the regulation of angiogenesis during development and pathological conditions. Prior research has established GPR182 as a negative regulator of definitive hematopoiesis in both mice and zebrafish [41]. Moreover, GPR182 expression extends beyond blood vascular ECs to include the liver sinusoidal endothelial cells (LSECs) and lymphatic ECs (LECs) [8, 25]. LSECs represent a specialized type of ECs characterized by fenestrations and the absence of a basement membrane in healthy hepatic tissue. GPR182 may be involved in maintaining the normal function of LSECs. During the progression of HCC, LSECs undergo multiple aberrant morphological and genetic changes. They lose their fenestrations and adopt a capillary phenotype, which leads to vascular dysfunction—a process termed LSEC capillarization [42]. The continuous ECs that replace the physiological LSECs in the liver exhibit elevated CD31 expression and reduced GPR182 expression, leading to a hypervascularized phenotype during tumorigenesis [43]. The potential roles of GPR182 in LSEC capillarization during HCC progression and in lymphangiogenesis are yet to be defined. Further investigations are required to expand the knowledge related the functions of GPR182 in these and other contexts.

Our findings, to our knowledge, mark the first demonstration of an EC-enriched GPCR serving as a negative regulator in angiogenesis. Pharmacological targeting of GPR182 could pave the way for innovative therapeutic strategies aimed at tumor-related and other disease-driven neoangiogenesis.

## Materials and methods

### Study approval

Experiments involving human samples were approved by the Affiliated Hospital of Nantong University (2020-K013). This study included 98 consecutive patients diagnosed with VTE, who admitted to the Affiliated Hospital of Nantong University between 2021 and 2023. The control group of the study included 62 healthy subjects, who were matched for age and gender with the study group. All participants signed informed written consent in this study and the investigation conformed to the principles outlined in the Declaration of Helsinki. All animal experimentation was carried out in accordance with NIH Guidelines for the care and use of laboratory animals (http://oacu.od.nih.gov/regs/index.htm) and ethically approved by the Laboratory Animal Center of Nantong University (NTULAC), Jiangsu Province, China (Approval ID: P20230228-043). Best efforts were made to minimize the number of animals used and prevent their suffering.

### Zebrafish strains, husbandry, anesthesia and euthanasia

The wild-type AB line and transgenic lines *Tg(fli1ep: EGFP-CAAX)*^*ntu666*^ [44], *Tg(gata1:DsRed)* [45], *Tg(flt1*^*BAC*^:*YFP)* [46], and *Tg(fli1a: nEGFP::kdrl: ras-mCherry)* [47] were used in this study. All zebrafish embryos and adult fishes were raised and maintained at 28.5ºC. Zebrafish embryos were anaesthetized using egg water containing 0.16 mg/ml tricaine (MS-222, Sigma) for live imaging. For euthanasia of zebrafish embryos, they were immersed in 300 mg/l tricaine for 10 min at 4 °C.

### Whole-mount in situ hybridization (WISH)

WISH with antisense RNA probes was performed as described previously [46]. Templates for making probes to detect the expression of *gpr182* (NM_001020478.1), *cxcr4a* (NM_131882.3), *vegfaa* (NM_001110349.2) and *esm1* (NM_001076741.1) were cloned from their corresponding cDNA fragments. The *gpr182* RNA probe was amplified with forward primer: 5’-CGAATCGCTCTTTTCCTTCTTTACC-3’ and reverse primer: 5’-GAAAATCAGGCATAGGAAGGAAACG-3’. The *cxcr4a* RNA probe was amplified with forward primer: 5’-TTTCTCCCAACGGTGTACGG-3’ and reverse primer: 5’-AGATCCATTTCTGCAGCCCC-3’. The *vegfaa* RNA probe was amplified with forward primer: 5’-TTATTTCTCGCGGCTCTCCT-3’ and reverse primer: 5’-ACAAGCGCTTCCTTCTCTCT-3’. The *esm1* RNA probe was amplified with forward primer: 5’-TTTTGGAGAGACTGAGGCGT-3’ and reverse primer: 5’-TGCTTTCAGTGTTGGTGTCG-3’. After hybridization, images of the embryos were acquired on an Olympus stereomicroscope MVX10 equipped with an Olympus DP71 camera.

### Morpholino-mediated gene knockdown of *gpr182* in zebrafish

The *gpr182* gene-specific morpholino (Gene Tools, LLC) was used to block the translation of *gpr182* mRNA. The sequence of the morpholino antisense oligomer was 5’-GTTGTGAATATCATGCGTCATGTTC-3’. The control MO was 5’-CCTCTTACCTCAGTTACAATTTATA-3’. In this study, 2 nL of 0.3 mM morpholino oligo was microinjected into the embryos at the 1-cell stage.

### mRNA rescue experiment

The cDNA fragment of *gpr182* was first amplified with a forward primer: 5’- CGGGATCCGCCACCATGACGCATGAATTCACAA − 3’ and a reverse primer: 5’- CGGAATTCTCATGCGCCAATGTCAGAGT − 3’, and subcloned into pCS2 + vector. DNA plasmid was then linearized with an appropriate restriction enzyme and transcribed in vitro using the SP6 mMessage mMachine kit (Ambion). In the rescue experiment, 200 pg *gpr182* mRNA was co-injected into the embryos with the *gpr182* antisense morpholino. The cDNA fragment of *gpr182* was first subcloned into pCS2 + vector. To make the mRNA, the DNA plasmid was linearized with appropriate restriction enzyme and transcribed in vitro using the SP6 mMessage mMachine kit (Ambion).

### Gene expression analysis by RNA-seq and real-time qPCR

Total RNAs of control and *gpr182*-MO injected zebrafish embryos at 36 and 48 hpf were respectively isolated using TRIzol Reagent (Invitrogen) and purified with the RNeasy Mini Kit (Qiagen) as recommended by the manufacturers. The RNA quality and quantity were measured using an Agilent 2100 Bioanalyzer (Agilent Technologies). One µg RNA was used for library preparation. The cDNA library preparation and next generation sequencing were performed according to the manufacturer’s protocol (Illumina). The sequences were processed and analyzed by GENEWIZ. Experiments were repeated 3 times and each replicate comprised of RNA extracted from 10 embryos. For real-time qPCR, cDNA synthesis was performed using the Transcriptor First Strand cDNA Synthesis Kit (Roche) according to the manufacturer’s instructions. All PCR amplifications were carried out using an ABI Prism 7000 thermocycler (Applied Biosystems). Gene expression data were normalized against *ef1α*. The primers for qPCR are listed below,

For the housekeeping gene *ef1a* (Accession: NM_131263.1),

Forward primer, 5′-TGATCTACAAATGCGGTGGA-3′;

Reverse primer, 5′-CAATGGTGATACCACGCTCA-3′.

For *cxcr4a* (NM_131882.3),

Forward primer, 5′-TGGCTTATTACGGACACATCGT-3′;

Reverse primer, 5′-CACATCACACGGGACCTCAA-3′.

For *vegfaa* (NM_001110349.2),

Forward primer, 5′-AAAAGAGTGCGTGCAAGACC-3′;

Reverse primer, 5′-GTTTCGTGTCTCTGTCGGGA-3′.

For *esm1* (NM_001076741.1),

Forward primer, 5′- AACCATGCGTGTGTTTGCCA-3′;

Reverse primer, 5′- CGGGCAATTCACGGCGTA-3′.

To study gene expression changes upon gpr182 knockdown, RNA-seq was conducted on prepared mRNAs from 36 and 48-hpf WT and *gpr182*-MO zebrafish embryos. Three independent replicates were analyzed for each treatment. All RNA samples were submitted to GENEWIZ Science (Suzhou, China), and deep sequencings were performed on an Illumina Hiseq2500 platform.

### Microangiography and confocal imaging

For confocal imaging of the vascular system, zebrafish embryos were treated with 1-phenyl-2-thiourea (Sigma-Aldrich) to inhibit the pigmentation. After manually dechorionation, embryos were anesthetized using egg water containing 0.16 mg/ml tricaine and 1% 1-phenyl-2-thiourea, and then embedded in 0.6% low melting agarose. For microangiography, 5 µg/µl Dextran TexasRed (Invitrogen) was injected into the common cardinal vein (CCV) at 72 hpf and the embryos were immediately processed for optical imaging. Living imaging was performed with Nikon A1R confocal microscopy.

### Zebrafish HCC model

The zebrafish HCC model was developed using the transgenic line *Tg(fabp10a: tetOn; tre: eGFP-kras*^*v12*^*)*, generously provided by Dr. Xu Wang from Fudan University. To facilitate in vivo monitoring of tumor angiogenesis, we created double transgenic zebrafish (*Tg(kdrl: ras-mCherry::fabp10a: tetOn; tre: eGFP-kras*^*v12*^*)*) by crossing the *Tg(fabp10a: tetOn; tre: eGFP-kras*^*v12*^*)* line with *Tg(kdrl: ras-mCherry)* line. Liver-specific expression of the oncogenic kras^v12^ was induced by administering 30 µg/mL Doxycycline (DOX) (Sigma Aldrich) starting from 3 dpf.

### Chemical treatment

Inhibition of the CXCL12-CXCR4 signaling axis was achieved using AMD3100 (Sigma-Aldrich). This compound was injected into the duct of Cuvier of gpr182 morphants at 24 hpf at a final concentration of 5 µM. For the HCC model, zebrafish larvae were treated with AMD3100 dissolved in egg water at a final concentration of 1 µM, starting at 2 dpi.

### Cell culture

HEK293T cells were maintained in DMEM medium containing 10% FBS. Lipofectamine™ 3000 Transfection Reagent (ThermoFisher Scientific) was used for plasmid transfection. The human GPR182 and CXCR4 cDNA were subcloned into pcDNA3-YFP oepcDNA3-CFP vectors (Addgene), and used for FRET assay or receptor expression. Human umbilical vein endothelial cells (HUVECs, CSC 2V0, Cell Systems) were cultured in endothelial cell growth medium according to the protocol provided by the manufacturer (VascuLife, Cell Systems). HUVECs at passage 3 were used. Lentiviruses expressing GPR182 shRNAs were transduced in HUVECs for gene knockdown. The GPR182 shRNA target sequence was 5’- GAGCTTTCAGGCACACCATTT-3’.

### Immunohistochemistry and immunofluorescence assay

Surgical specimens of HCC tissues were fixed in formalin and embedded in paraffin. The sections were stained with anti-human GPR182 (Abcam) using a standard indirect avidin-biotin HRP method. For immunofluorescence assay, slides were labeled with anti-CD31 and anti-GPR182 antibodies, followed by the appropriate secondary antibodies. Cells were seeded in 15-mm glass bottom cell culture dishes (Corning). After a day, the cells were washed three times with PBS and fixed with 4% paraformaldehyde for 20 min. The cells were then washed three times with PBS, and permeabilized with 0.2% Triton X-100 (Beyotime) at room temperature for 10 min. After washing three times with PBS, the cells were incubated overnight at 4 °C with the primary anti-GPR182 or anti-CXCL12 or anti-Rab5 antibodies (Abcam). After washing three times with PBS, and incubating with a fluorescent dye-labelled secondary antibody for 1 h.

### Live-cell imaging for GPCR downstream signaling analysis

A quantitative live-cell imaging system developed by Tany et al. for detecting GPCR downstream signaling was utilized [26]. This system employed two stable HeLa cell lines: one expressing the cAMP biosensor and R-GECO1.0 (HeLa/cAMP/Ca^2+^), and the other expressing DORA-RhoA and ERK-KTR (HeLa/RhoA/ERK), along with linker histone H1-mCherry as a nuclear marker to measure the cytoplasm-to-nucleus (C/N) ratio. These cell lines were co-cultured and transfected with plasmids encoding GPCR and a puromycin-resistance gene. Post-transfection, cells were selected for GPCR expression using puromycin. Following selection, cells were serum-starved for approximately 18 to 25 h to minimize basal ERK activity before imaging.

### Förster resonance energy transfer (FRET) assay

FRET was measured by photobleaching in HEK293T cells transiently cotransfected with CXCR4-CFP/GPR182-YFP or CXCR4-CFP/CXCR4-YFP or GPR182-CFP/GPR182-YFP. Photobleaching of YFP was done using 514-nm excitation over time periods that lasted up to 30 s. CFP and YFP signals before and after photobleaching were separately acquired with Nikon A1R confocal microscopy. FRET efficiency calculated in percentage as E = [(*I*_*CFP−After*_ - *I*_*CFP−Before*_)/*I*_*CFP−After*_] X 100, where *I*_*CFP−Before*_ and *I*_*CFP−After*_ are the background-corrected CFP fluorescence intensities before and after YFP photobleaching, respectively. CXCR4-CFP/CXCR4-YFP and GPR182-CFP/GPR182-YFP were used as positive and negative control, respectively.

### Statistical analysis

Statistical analysis employed a two-tailed, unpaired Student’s t-test or One-way ANOVA, is described in figure legends. All data are presented as mean ± SD. *P* < 0.05 was considered to be statistically significant.

## Electronic supplementary material

Below is the link to the electronic supplementary material.

Supplementary Material 1

## Data Availability

The data that support the findings of this study are available from the corresponding author upon reasonable request.
