# Supplementary material for: G protein-coupled receptor GPR182 negatively regulates sprouting angiogenesis via modulating CXCL12-CXCR4 axis signaling
Source: Angiogenesis. 2025 May 2;28(3):25. doi: 10.1007/s10456-025-09977-5 (PMC12048421; doi:10.1007/s10456-025-09977-5)
Supplement: Supplementary file 1 — Supplementary Material 1 [file 10456_2025_9977_MOESM1_ESM.docx]

**Title:** G protein-coupled receptor GPR182 negatively regulates sprouting angiogenesis via modulating CXCL12-CXCR4 axis signaling

**Running title:** Role of GPR182 in angiogenesis

Changsheng Chen^1,*,#^, Wei Liu^1,6,7,*^, Fang Yuan^2,8,*^, Xiaoning Wang^3^, Xi Xu^2^, Chang Chun Ling^4^, Xiaojuan Ge^1^, Xiaozhong Shen^2,5^, Bowen Li^1^, Yuqian Shen^1,9^, Dong Liu^1,2,10,#^

^1^School of Life Sciences, Nantong Laboratory of Development and Diseases; Nantong University, Nantong, Jiangsu Province, China

^2^Medical College of Nantong University, Nantong, Jiangsu Province, China

^3^Research Center of Clinical Medicine, Affiliated Hospital of Nantong University, Nantong, Jiangsu Province, China

^4^Department of Intervention and Vascular Surgery, Affiliated Hospital of Nantong University, Nantong, Jiangsu Province, China

^5^State Key Laboratory of Medical Genomics, Shanghai Institute of Hematology, National Research Center for Translational Medicine, Ruijin Hospital affiliated to Shanghai Jiao Tong University School of Medicine, Shanghai, China

^6^The State Key Laboratory Breeding Base of Basic Science of Stomatology & Key Laboratory of Oral Biomedicine Ministry of Education, School & Hospital of Stomatology, Medical Research Institute, Wuhan University, Wuhan, Hubei Province, China

^7^Frontier Science Center for Immunology and Metabolism, Wuhan University, Wuhan, Hubei Province, China

^8^Huai’an TCM Hospital Affiliated to Nanjing University of Chinese Medicine, Huai’an, Jiangsu Province, China

^9^Department of Translational Medicine, IGBMC, INSERM U964, CNRS UMR7104, Université de Strasbourg, Illkirch, France

^10^Key Laboratory of Neuroregeneration of Jiangsu and Ministry of Education, Co-innovation Center of Neuroregeneration, Nantong University, Nantong, Jiangsu Province, China

*, these authors contributed equally to this work

#, authors for correspondence

Contact details for Correspondence:

**Dong Liu**, Ph.D

School of Life Sciences, Nantong Laboratory of Development and Diseases, Nantong University, Seyuan Road 9, Nantong, China, 226019

Phone: + (86)-18605133927; Fax: + (86)-513-85012809

Email: [liudongtom@gmail.com](mailto:liudongtom@gmail.com); [tom@ntu.edu.cn](mailto:tom@ntu.edu.cn)

**Changsheng Chen**, Ph.D

School of Life Sciences, Nantong Laboratory of Development and Diseases, Nantong University, Seyuan Road 9, Nantong, China, 226019

Phone: + (86)-18851428272

Email: [c.chen@ntu.edu.cn](mailto:c.chen@ntu.edu.cn)

**
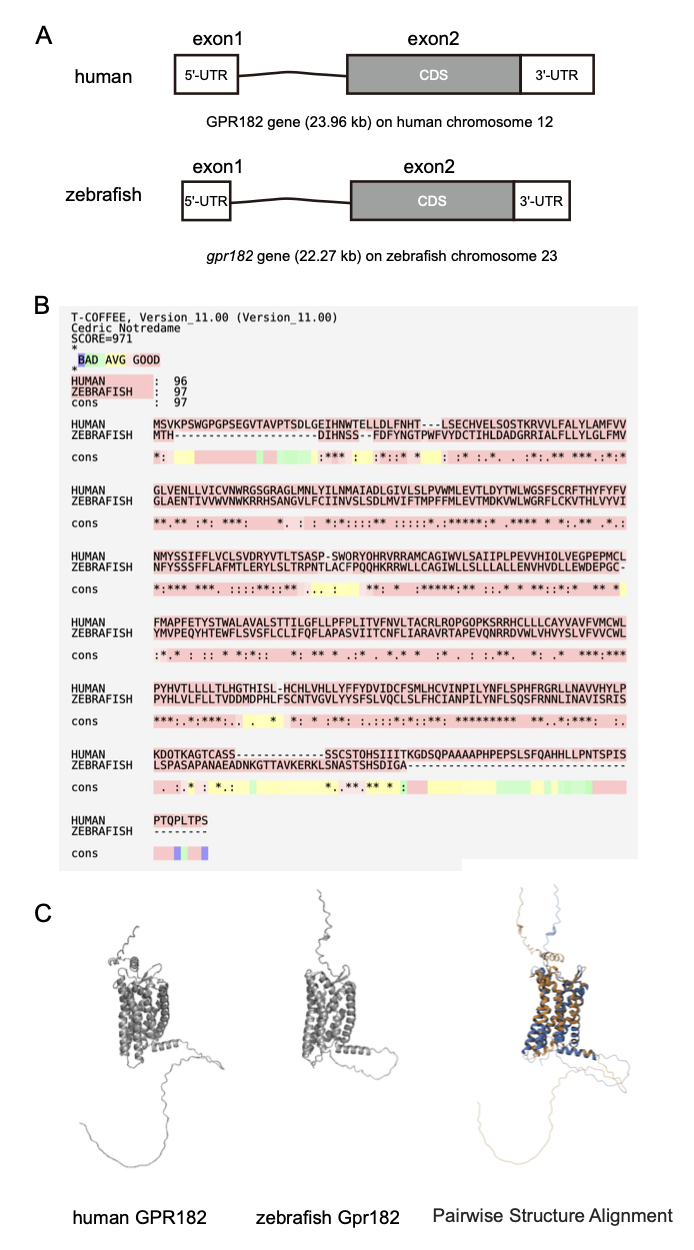
**

**Supplementary Figure 1. GPR182 is highly conserved between human and zebrafish. (A)** Schematic representation of *GPR182* gene structure in human and zebrafish. **(B)** Alignment of GPR182 protein sequence between human and zebrafish. The multiple sequence alignment result is produced by T-coffee and modified with JalView software. **(C)** Pairwise structural alignment of human and zebrafish GPR182, generated by Alphafold2, further highlighting the conservation at the structural level.


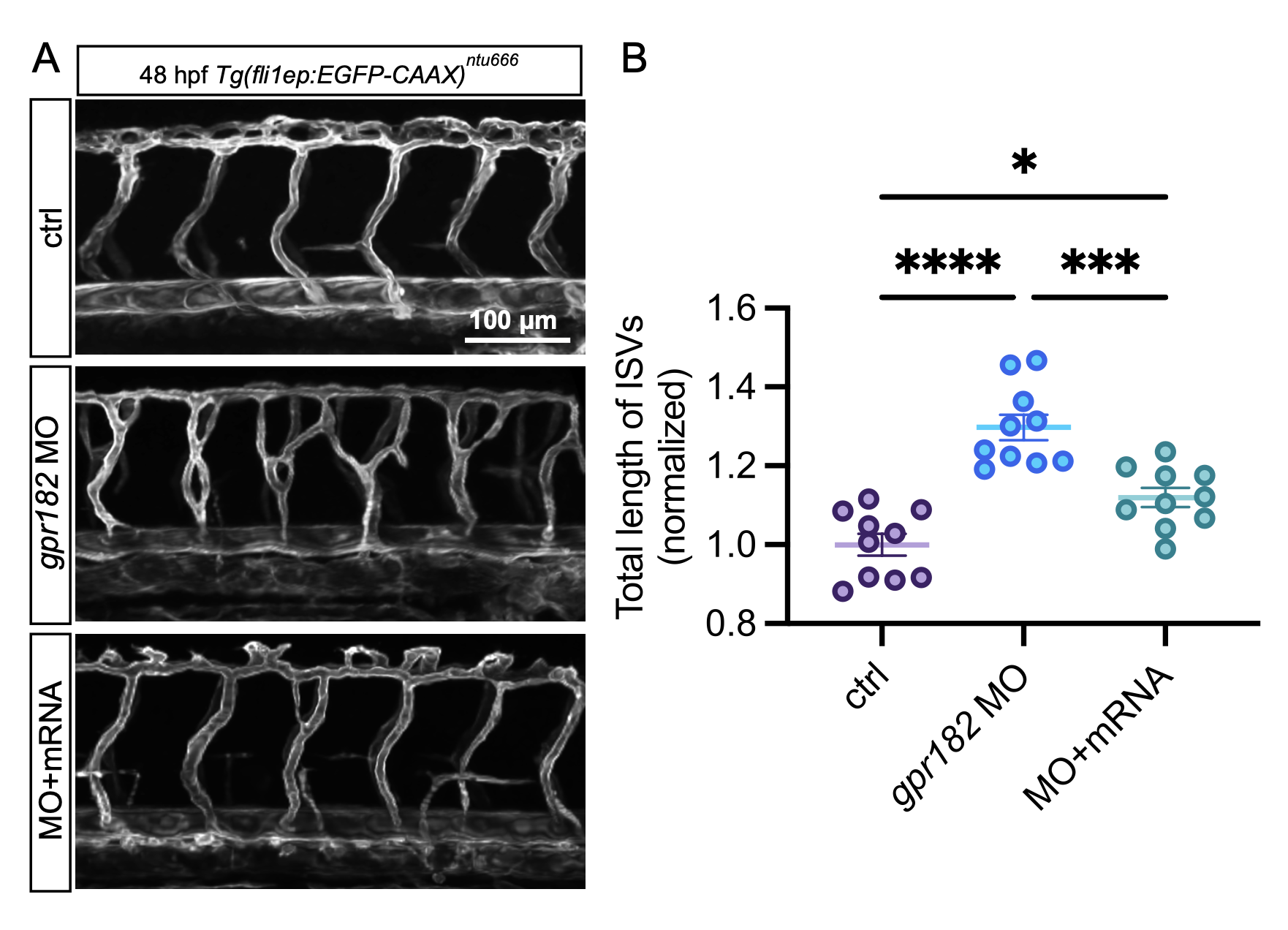


**Supplementary Figure 2. Overexpression of *gpr182* rescues the vascular phenotype of the morphants. (A)** Confocal images of vascular morphology in *Tg(fli1ep:EGFP-CAAX)^ntu666^* control embryos, embryos injected with *gpr182* MO, and embryos injected with *gpr182* MO and *gpr182* mRNA at 48 hpf. Scale bars, 100 μm. **(B)** Quantitative analysis of total ISV length. Data are presented as mean ± SD, with statistical differences determined by one-way ANOVA. ***, p < 0.001; ****, p < 0.0001.


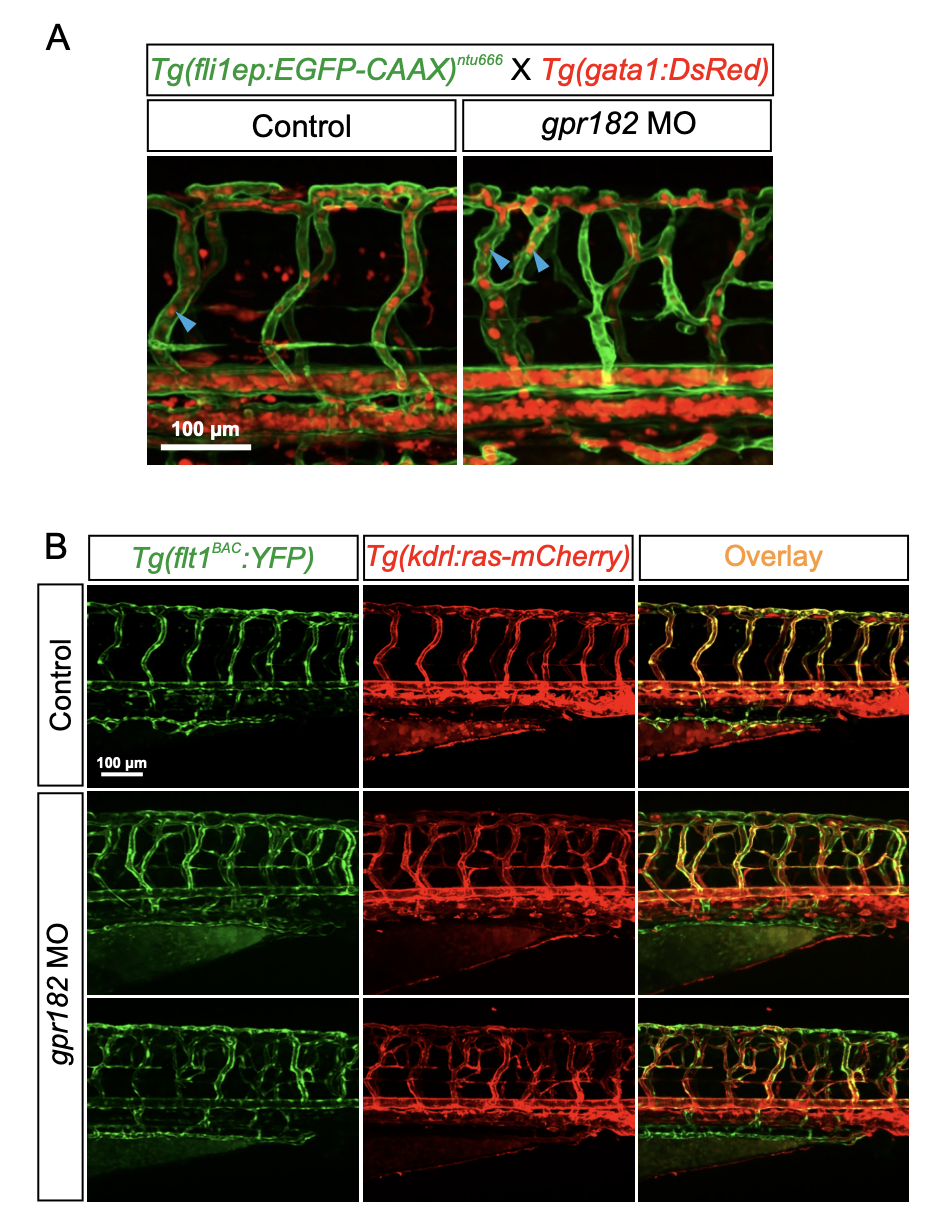


**Supplementary Figure 3. Excessive vascular branches in *gpr182* morphants are functionally perfused. (A)** Confocal images of *Tg(fli1ep:EGFP-CAAX::gata1:DsRed)* double transgenic embryos show that the hyperbranched vessels in *gpr182* morphants are perfused with red blood cells, indicated by blue arrowheads. (B) Confocal images of *Tg(flt1^BAC^:YFP::kdrl:ras-mCherry)* double transgenic embryos verifies the hyperbranching phenotype caused by Gpr182 deficiency.
